# Effect of Multiwalled Carbon Nanotubes on the Mechanical Properties of Carbon Fiber-Reinforced Polyamide-6/Polypropylene Composites for Lightweight Automotive Parts

**DOI:** 10.3390/ma11030429

**Published:** 2018-03-15

**Authors:** Huu-Duc Nguyen-Tran, Van-Tho Hoang, Van-Ta Do, Doo-Man Chun, Young-Jin Yum

**Affiliations:** School of Mechanical Engineering, University of Ulsan, Ulsan 44610, Korea; huuducqs@gmail.com (H.-D.N.-T.); hoangvantho.ntu@gmail.com (V.-T.H.); vantado.ntu@gmail.com (V.-T.D.); dmchun@ulsan.ac.kr (D.-M.C.)

**Keywords:** multiwalled carbon nanotubes, carbon fiber, thermoplastic polymer, PP, PA6, composites, lightweight

## Abstract

The development of lightweight automotive parts is an important issue for improving the efficiency of vehicles. Polymer composites have been widely applied to reduce weight and improve mechanical properties by mixing polymers with carbon fibers, glass fibers, and carbon nanotubes. Polypropylene (PP) has been added to carbon fiber-reinforced nylon-6 (CF/PA6) composite to achieve further weight reduction and water resistance. However, the mechanical properties were reduced by the addition of PP. In this research, multiwalled carbon nanotubes (CNTs) were added to compensate for the reduced mechanical properties experienced when adding PP. Tensile testing and bending tests were carried out to evaluate the mechanical properties. A small amount of CNTs improved the mechanical properties of carbon fiber-reinforced PA6/PP composites. For example, the density of CF/PA6 was reduced from 1.214 to 1.131 g/cm^3^ (6.8%) by adding 30 wt % PP, and the tensile strength of 30 wt % PP composite was improved from 168 to 173 MPa (3.0%) by adding 0.5 wt % CNTs with small increase of density (1.135 g/cm^3^). The developed composite will be widely used for lightweight automotive parts with improved mechanical properties.

## 1. Introduction

Recently, the use of lightweight materials has become an important strategy in the automotive field because the weight reduction of automotive parts can improve fuel efficiency and reduce carbon dioxide emissions [1,2,3]. Lightweight material substitution is a preferred approach for improving fuel efficiency without significantly changing the vehicle size or components. In addition, lightweight materials can improve acceleration, ride, and handling as well as reducing noise. Electric vehicles must particularly focus on vehicle weight in order to save energy and minimize the motor power and battery requirements. Additional components (such as batteries) may be added due to the weight reduction of structural parts, and the range of vehicles can therefore be extended while keeping the same design [4]. The main advantages of composite materials, as compared with conventional heavy metals, are lower density, high specific strength, and high specific stiffness. 

Lightweight materials such as aluminum, magnesium, plastics, and composites have replaced heavy metals in vehicles [5,6]. Among them, carbon fiber-reinforced plastics (CFRP) are a practical solution for weight reduction in vehicles. Compared to glass fibers, carbon fibers (CF) have a lower density and higher strength and modulus with excellent thermal and electrical conductivity; this makes them attractive for many applications, especially in the automotive industry [7,8,9]. Thermosetting resins are widely used as matrix materials. However, they cannot be melted and reshaped after curing, and recycling thermosetting resins is extremely difficult. On the other hand, thermoplastic matrix materials are convenient for recycling and mass production using injection molding, which allows designers to design the products they desire in terms of shape and structure [10]. Among thermoplastic polymers, nylon 6 or polyamide 6 (PA6) shows good mechanical properties and is used widely as an engineering plastic. However, PA6 can easily absorb water, and the mechanical properties of PA6 are significantly affected by the absorption of water. It was reported that the tensile strength of PA6 composites decreases significantly after storage in water [11,12]. To reduce water absorption and decrease the effects on mechanical properties under humid conditions, PA6 is frequently blended with polypropylene (PP), which provides good resistance against moisture and ensures good processability [13]. 

PP is inexpensive and has low density, and blending PP with PA6 composites can reduce both the material cost and the density. However, PP and PA6 are incompatible because of their different polarities and crystalline morphologies, so the use of a compatibilizer is required to enhance the miscibility of the blend. A typical compatibilizer is maleic anhydride-grafted polypropylene (MaPP) [12,13,14]. PP blending with PA6 composites can result in weight reduction, as well as improve the mechanical properties during water absorption. Thus, this strategy can be used in automotive exterior parts in the rainy season or in underwater applications. However, carbon fiber-reinforced PA6/PP composites showed relatively lower mechanical properties than carbon-reinforced PA6 under dry conditions. A high weight ratio of CF can be used to increase the mechanical properties of CFRP, but the density, viscosity, and brittleness then increase [15]. 

Carbon nanotubes (CNTs) have been widely used as a filler material for composites due to their superior mechanical and physical properties [16,17]. Solution blending, melt blending, and in situ polymerization have been widely applied for fabricating carbon nanotube (CNT)/polymer composites to make CNT-reinforced composites with improved mechanical properties [18]. In addition, magnetically aligned CNTs were used in CF polymer composites, and the flexural modulus and load carrying capacity were increased by 46% and 33%, respectively [19]. CNTs were also directly grown onto carbon fiber surfaces using an aerosol-assisted chemical vapor deposition method to reinforce epoxy composites; this resulted in a 94% improvement in interfacial shear strength as measured using single fiber pull-out tests of micro-droplet composites, while the tensile strength slightly decreased within 10% [20]. However, simple CNT mixing without any additional treatments of CNTs are preferred due to its reduced process time and cost for mass production. Moreover, CNTs have been successfully used to reinforce nylon-6 composites [21,22] and nylon-6/polypropylene composites [23] without any treatment processes. However, to the best of our knowledge, there are not any studies about the effect of CNTs on CF-reinforced PA6/PP composites by simple melt blending for simple industrial applications. 

In this research, CNTs were evaluated as additional fillers for CF-reinforced PA6/PP composites to improve the mechanical properties and minimize the increase in density of the composite. Composites with different CNT ratios from 0.5, 1, and 1.5 wt % were synthesized by simple melt blending, which can be easily used in industry, with commercially available PA6, PP, MaPP, CF, and CNTs without any treatments, and specimens for the testing of mechanical properties were fabricated by injection molding. Tensile testing and bending tests were carried out to evaluate the mechanical properties. A small amount of CNTs improved the mechanical properties of carbon fiber-reinforced PA6/PP composites, and the density increase was minimized.

## 2. Experiment

### 2.1. Sample Preparation

**Figure 1:**
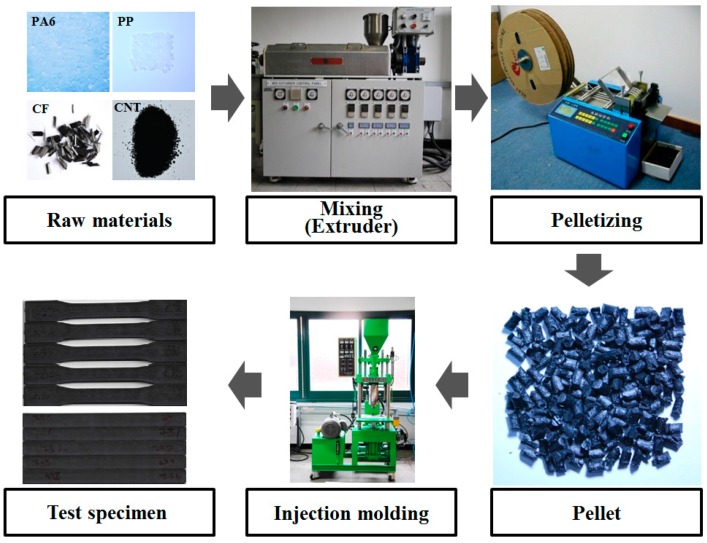
Sample preparation procedure.

Commercially available PA6 (KN111, Kolon Plastics, Gwacheon, Korea), PP (SJ-170, Lotte Chemical, Seoul, Korea), CF (CFU 6m, Nippon Polymer Sangyo, Osaka, Japan), MaPP (G-3003, Eastman Chemical Company, Kingsport, TN, USA), and CNTs (CM-130, Hanwha Chemical, Seoul, Korea) were used as purchased without any treatments. CF is chopped carbon fiber with urethane sizing agent, 6 mm of cutting length and 7 μm of fiber diameter. CNTs were produced by chemical vapor deposition (CVD), with a purity of about 90 wt % (86–92 wt %) and a bulk density in the range of 0.025 to 0.050 g/cm^3^. Melt blending and injection molding were used for sample preparation. The sample preparation procedure is summarized in Figure 1. First, the polymer materials were dried for 2 h in an oven at 80 °C, and then mixed with different mixing ratios of raw materials before being fed into the melt blending machining. In this research, the mixing ratio of CF was fixed at 20 wt % (about 9~14 vol %), and the mixing ratios of CNTs were 0, 0.5, 1, and 1.5 wt %. The remaining material was a PA6/PP blend with a PP concentration of 0, 10, 20, or 30 wt %. For PA6/PP blending, MaPP was used to enhance the miscibility of the blend. The mixing ratio of MaPP was 5 wt % of the weight of PP, as per the manufacturer’s guidelines. The mixing ratios are summarized in Table 1.

Melt blending was carried out twice using a single screw extruder (Seawon M-Tech, Gunpo, Korea). Pellets (length and radius were around 5 and 3 mm, respectively) were produced using a cutting machine. Finally, specimens for tensile testing and flexural testing were fabricated using an injection molding machine (Wonil Hydraulic Co., Ltd., Anyang, Korea). The specimen shape for the tensile test was type I of ASTM D-639, and the specimen dimensions for the flexural test were 165 mm × 12.7 mm × 3.2 mm according to ASTM D-790-3. The process parameters for the single screw extruder and the injection molding machine are listed in Table 2.

### 2.2. Evaluation Methods

Mechanical properties were evaluated by tensile tests and flexural tests. The specimens were stored in a sealed plastic bag just after fabrication to minimize water absorption, and at room temperature and a relative humidity lower than 40%. Tensile and flexural tests were carried out with five specimens of each composite with different compositions. Tensile tests were carried out on a Universal Tensile Machine (Daekyung Tech and Testers, Incheon, Korea) at a constant cross-speed of 5 mm/min, according to ASTM D-639, with a 50-mm gauge length. Flexural tests were performed in the three-point loading mode using a Flexural Machine Test (Eun Sung Machine, Busan, Korea) at a crosshead speed of 1.62 mm/min according to ASTM D790-3. After the tensile test, the fractured surface was observed using a field emission scanning electron microscope (FE-SEM, Jeol Co., Tokyo, Japan). The fractured surface was coated by Pt for FE-SEM. The density of the specimen was measured according to Archimedes’ principle using a balance with 0.001 g precision. To investigate the dispersion of PP in the composite matrix, the fracture surface of the specimen was treated in hot xylene to remove the PP, and the surface was observed by FE-SEM.

## 3. Results

The mechanical properties and densities of the CF-reinforced PA6/PP composites without CNTs are summarized in Table 3. The values are average values and standard deviations of five samples for each weight fraction of PP.

The mechanical properties of composite materials change according to the rule of mixtures. The effect of each material is strongly dependent on its volume (or weight) fraction. The addition of PP reduced the mechanical properties of the composite due to the poor mechanical properties of PP and its incompatibility with PA6/PP [12]. However, the bonding was improved by MaPP [24]. In this research, the mechanical properties (tensile strength, elastic modulus, flexural strength, and flexural modulus) of the CF-reinforced PA6/PP composites without CNTs decreased as the weight fraction of PP increased. The density of PP (0.90 g/cm^3^) is much lower than the density of PA6 (1.14 g/cm^3^). The densities of the composites were reduced as the weight fraction of PP increased. The CF-reinforced PA6/PP composite with 30 wt % PP was lighter than PA6, so additional weight reduction may be possible with better mechanical properties than those provided by pure PA6. In addition, water resistance was also improved, and the results were reported in our previous paper [25]. After water saturation, the tensile strength and elastic modulus of the CF-reinforced PA6 without PP became smaller than those of CF-reinforced PA6/PP composite with 30 wt % PP.

**Figure 2:**
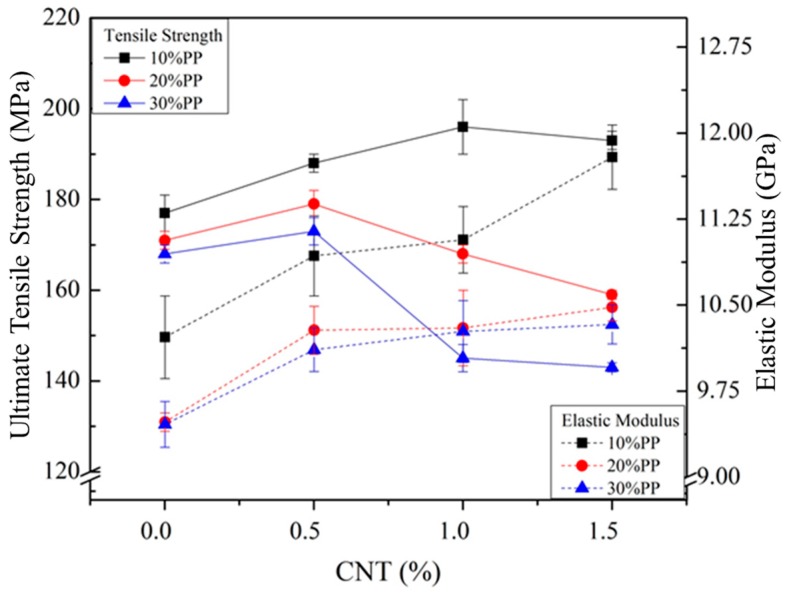
Tensile strength and elastic modulus results of CF- and CNT-reinforced PA6/PP composites with different PP and CNT compositions.

**Figure 3:**
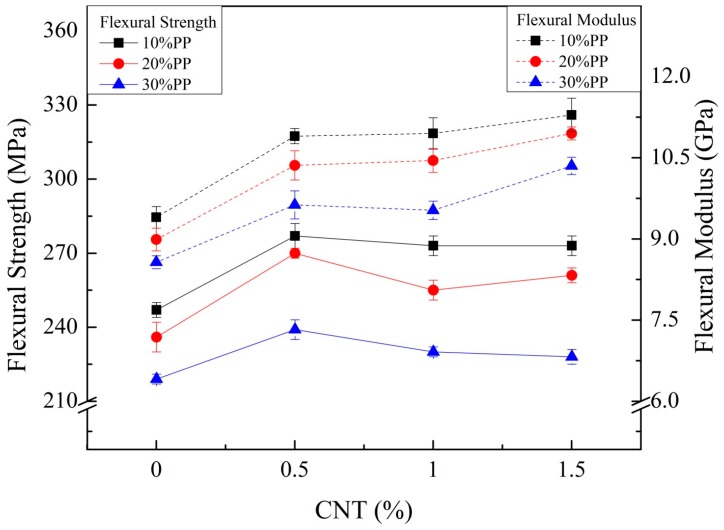
Flexural strength and modulus results of CF- and CNT-reinforced PA6/PP composites with different PP and CNT compositions.

**Figure 4:**
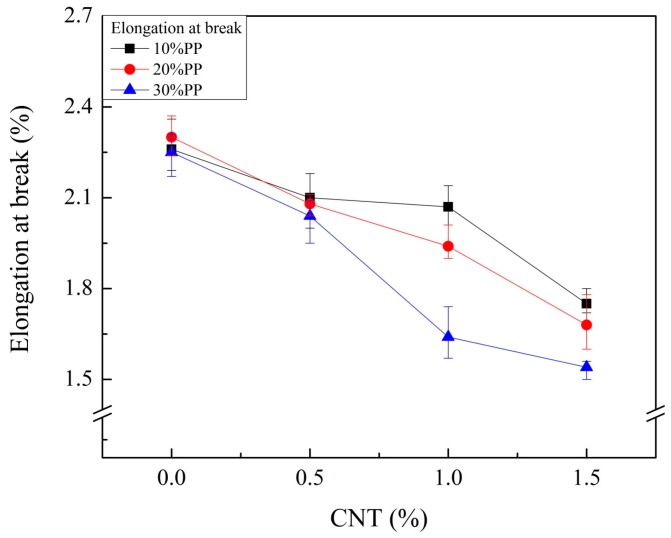
Elongation at break results of CF- and CNT-reinforced PA6/PP composites with different PP and CNT compositions.

Additional weight reduction and good water resistance were achieved by blending PP with CF-reinforced PA6 composite. However, the mechanical properties were reduced. A small amount of CNTs was added to compensate for the reduced mechanical properties. Figure 2 and Figure 3 show the tensile and flexural properties of composites with different weight fractions of CNTs. Both elastic and flexural moduli increased as the weight fraction of CNTs increased, and the maximum values were observed with 1.5 wt % of CNT. The tensile and flexural strength showed maximum values with a certain weight fraction (0.5 or 1.0 wt %) of CNT. After that point, they decreased as the weight fraction of CNTs increased. Thus, a small amount of CNTs can improve the mechanical properties of CF-reinforced PA6/PP composites as an additional reinforcing material, but a large amount of CNTs can show the opposite effect on strength. Furthermore, the elongations at break of the composite were also affected by CNTs. Elongations at break decreased as the weight fraction of CNTs increased, as shown in Figure 4. A possible reason for this is the restricted chain mobility of polymer molecules due to the increased amount of CNTs [26].

**Figure 5:**
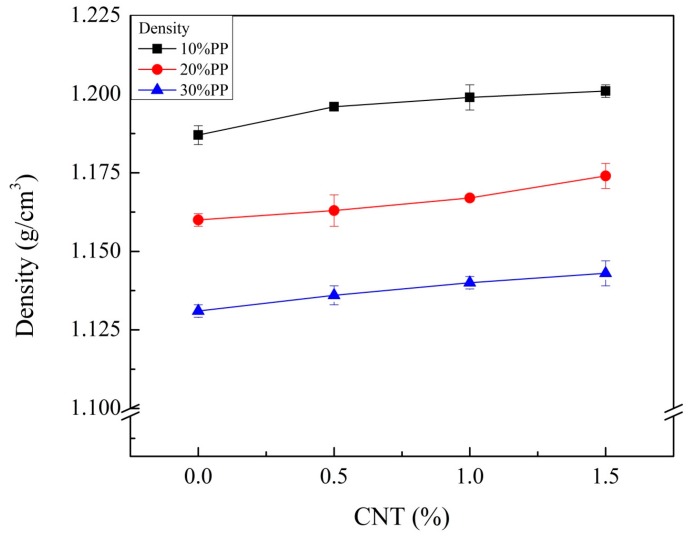
Density results of CF- and CNT-reinforced PA6/PP composites with different PP and CNT compositions.

PP plays an important role in the weight reduction of composites. However, CNTs could increase the composite density. Figure 5 shows the densities of the CNT-reinforced composites. The densities showed an almost linear relationship between the composite density and weight fraction of CNT, but the density of the composite slightly increased with increasing CNT composition. Thus, PP had a dominant effect on density.

A small amount of CNTs can improve the mechanical properties such as strength and modulus, but elongation at break can be reduced. Even though CNTs can increase the density, the effect was very small because of the small amount of CNTs.

## 4. Discussion

**Figure 6:**
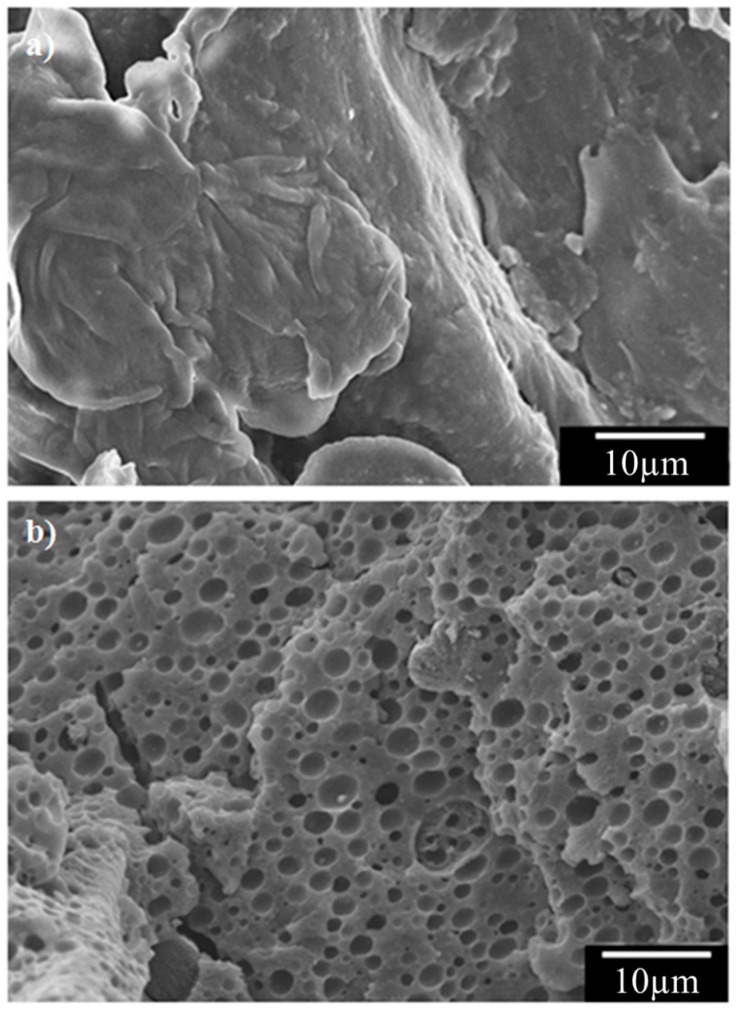
FE-SEM images of the fractured surfaces of CF-reinforced PA6/PP composites with (**a**) 0% PP and (**b**) 30% PP after etching PP by a xylene treatment.

To confirm the dispersion of PP in the composite, composites of 0% PP and 30% PP without CNTs (0% PP + 0.0% CNT and 30% PP + 0.0% CNT) were treated by hot xylene to etch the PP phase on the fractured surface of the specimens. The 30% PP + 0.0% CNT composite showed many small holes with diameters of less than 5 μm (Figure 6b), while there were no holes on the fractured surface of the 0% PP + 0.0% CNT composite (Figure 6a). The holes in the 30% PP + 0.0% CNT composite were PP, and large PP particles were not observed. Therefore, the PP was uniformly dispersed as particles with diameters of less than 5 μm in the PA6 matrix.

**Figure 7:**
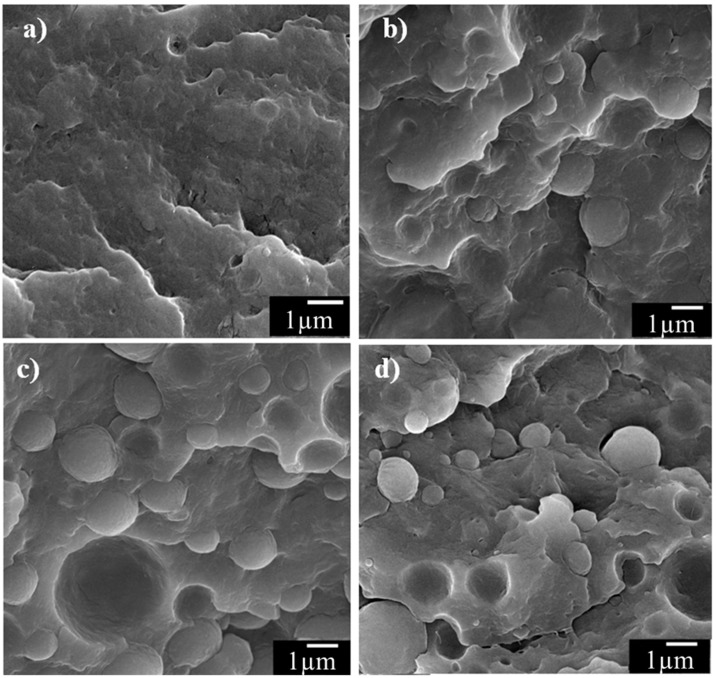
FE-SEM images of the fractured surfaces of CF-reinforced PA6/PP composites with (**a**) 0 wt %, (**b**) 10 wt %, (**c**) 20 wt %, and (**d**) 30 wt % PP without CNTs.

The fractured surfaces of CF-reinforced PA6 composites with different weight fractions of PP without CNTs (0% PP + 0.0% CNT, 10% PP + 0.0% CNT, 20% PP + 0.0% CNT, and 30% PP + 0.0% CNT) were observed, as shown in Figure 7. The fractured surface without PP (0% PP + 0.0% CNT) showed fracture of the matrix (Figure 7a), but the fractured surfaces with PP (10% PP + 0.0% CNT, 20% PP + 0.0% CNT, and 30% PP + 0.0% CNT) showed clear separation of the interfaces between PA6 and PP (Figure 7b–d). Based on Figure 7b–d, the PP phase was clearly separated from the PA6 matrices, and the holes and knobs on the fractured surface of the composites increased as the weight fraction of PP increased. This result indicated that the bonding between PA6 and PP was weaker than the strength of PP and PA6, so clear holes and knobs structures were observed on the fractured surfaces. Similar dispersed PP phases in matrices with knobs and holes were previously reported, and discontinuities and boundaries of the PP phase were considered to be defects in the matrix [23]. Therefore, the reductions in strength and modulus with increased PP composition can be explained in terms of the weak mechanical properties of PP and the weak bonding between PA6 and PP.

**Figure 8:**
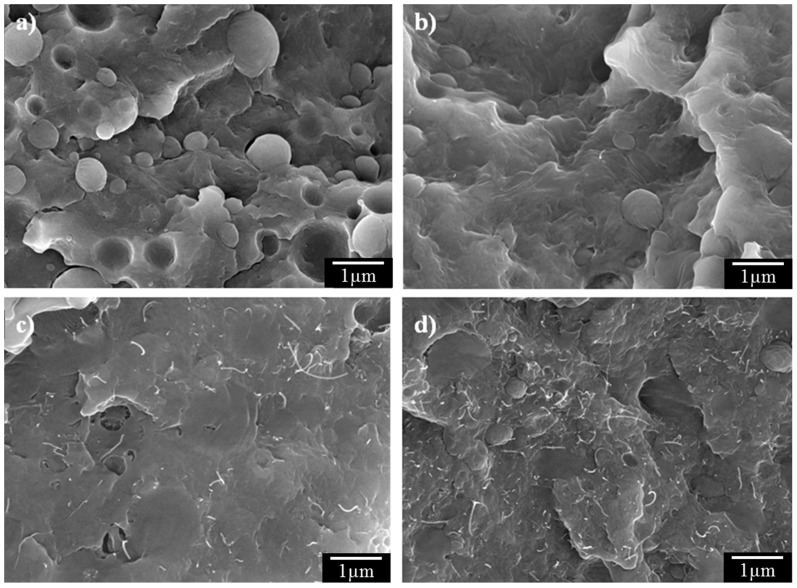
FE-SEM images of the fractured surfaces of CF-reinforced PA6/PP composites (30 wt % PP) with (**a**) 0 wt %, (**b**) 0.5 wt %, (**c**) 1 wt %, and (**d**) 1.5 wt % CNTs.

However, a small amount of CNTs could increase the strength and modulus. This is evident from the fractured surfaces of 30% PP composites with different CNT compositions (30% PP + 0.5% CNT, 30% PP + 1.0% CNT, and 30% PP + 1.5% CNT), shown in Figure 8. Clear separation of PP and PA6 on the 30% PP composite without CNTs (30% PP + 0.0% CNT) was observed (Figure 8a). In contrast, the CNTs with 30% PP (30% PP + 0.5% CNT, 30% PP + 1.0% CNT, and 30% PP + 1.5% CNT) showed clear matrix fracture, and the number of holes and knobs on the fractured surfaces was clearly reduced (Figure 8b–d). The bright dots or tubes indicate CNTs in Figure 8b–d, and the CNT fibers, which were pulled out of the fractured surface, increased as the CNT weight fraction increased. CNTs were also used as reinforcing materials, and the elastic and flexural moduli increased as the weight fraction of CNTs in the composite increased due to randomly oriented CNTs. The strength of the composite depended on the number of CNTs oriented in the same loading direction as that in the composite. Other fillers also contributed to the tensile modulus and flexural modulus. According to Chow et al. [27], nano organo-clay filler in a composite was reported to have a random distribution except in the near-skin area of the specimen in PA6/PP (70/30 wt %) composites, which were fabricated using an injection molding method.

**Figure 9:**
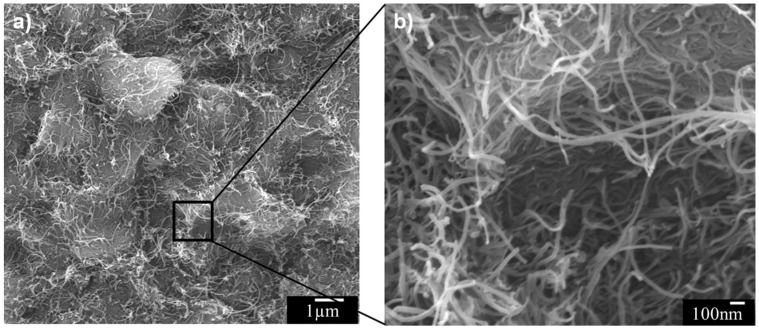
FE-SEM images of (**a**) CNT agglomeration in the fracture surface of CF-reinforced PA6/PP composites (30 wt % PP) with 1.5 wt % CNTs and (**b**) its enlarged image.

Furthermore, agglomerates of CNTs in a composite with relatively large weight fractions of CNTs were easily produced in the matrix, which may decrease the mechanical properties if they act as defects [17]. In this research, entangled CNT agglomerates were found in the composite with 1.5 wt % CNTs. Agglomerates of CNTs were easily found on the fracture surfaces of the 30% PP + 1.5% CNT composite, as shown in Figure 9. Figure 8d and Figure 9 were obtained from the same fractured sample, and the morphologies of these figures illustrate a clear difference. This result can explain the reason why the tensile and flexural strengths were reduced with a high weight fraction of CNTs. Agglomerates of CNTs cannot reinforce the matrix, and the CNTs can be separated easily. The CNT ratio of 1.5 wt % is relatively low to have agglomeration when an appropriate process is involved. However, this research tried to use conventional melt blending for material preparation without any additional processes for CNT dispersion except simple melt blending. Additional CNT dispersion processes or surface treatment of CNTs for dispersion can solve this problem [28]. However, the composite prepared with simple melt blending can show the agglomeration of CNTs, which can in turn have a negative effect on the mechanical properties of the composite.

**Figure 10:**
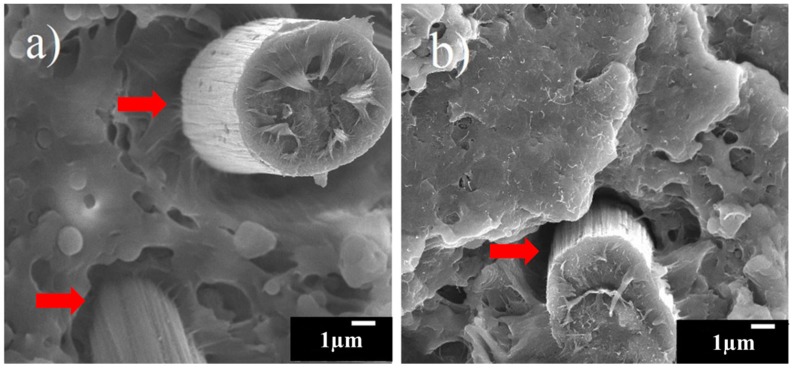
FE-SEM images of the interfaces between CF and the matrix of 30% PP composite on the fractured surface (**a**) without CNTs and (**b**) with 1 wt % CNTs.

The interface of the CF and matrix strongly influences the mechanical properties of the composites [29,30]. It was reported that fiber degradation increased with increasing CNTs due to filler-fiber interactions via a simple melting method [15,31]. The filler-fiber interactions made the fibers shorter, which may be one of the reasons for the reduced mechanical properties found in this research. In addition, the interfaces between CF and the matrix can be affected by CNT content. According to Feng et al. [32], the contact angle of epoxy resin droplets with different CNT contents from 0 to 1 wt % on carbon fiber bundles showed the lowest content, with 0.5 wt % of CNT. Low contact angle means large attraction force between CF- and CNT-mixed resin, and the transverse tensile strength, which can show the bonding between CF- and CNT-mixed resin, showed the same trend as the contact angle results. The transverse tensile strength was increased as the CNT content increase up to 0.5 wt %, but the strength was decreased with further increasing the CNT content. The tensile strength and flexural strength results in this research showed similar trends. In addition, different bonding morphologies of the CF and matrix with different CNT contents were observed at the interface between the CF and matrix, as shown in Figure 10. With a relatively large amount of CNTs, separation of the interfaces between the CF and matrix was often observed because CNTs on the CF can reduce the adhesion between the CF and matrix with a relatively large amount of CNTs.

The results suggest that further weight reduction may be possible by adding a small amount of CNTs without reducing the mechanical properties of CF-reinforced PA6/PP. Table 4 shows a comparison of the mechanical properties and densities of CF-reinforced PA6/PP composites without CNTs and with CNTs. The mechanical properties of CF-reinforced PA6/PP composites with reduced densities were improved by adding a small amount of CNTs via simple melt blending.

## 5. Conclusions

PP has been added to CF-reinforced PA6 composite to achieve further weight reduction and water resistance, which is especially useful for exterior automotive parts that will be exposed to rain. For further weight reduction and improved water resistance of the PA6/CF composite, PP blending was one option that showed good performance, especially for high humidity or underwater applications. However, the ultimate tensile strength, elastic modulus, and elongation of the composites decreased with increasing PP without water absorption. In this research, the effect of CNTs as additional fillers for CF-reinforced PA6/PP composites was evaluated to improve the mechanical properties and minimize the density of the composite. Composites with different CNT ratios (0.5, 1, and 1.5 wt %) were synthesized by simple melt blending with a single screw extruder, which a typical material mixing process in the industry, with commercially available PA6, PP, MaPP, CF, and CNTs without any additional treatments in order to reduce process time and cost for mass production. Specimens for mechanical property testing were fabricated by injection molding. Tensile tests and bending tests were carried out to evaluate the mechanical properties. Further weight reduction and improved mechanical properties similar to those of CF-reinforced PA6 composites were achieved by blending CNTs and PP. The drop in mechanical properties of CF-reinforced PA6/PP composites with reduced densities was compensated by adding a small amount of CNTs via simple melt blending. However, a relatively large amount CNTs reduced the mechanical properties due to the formation of agglomerates of CNTs and fiber degradation, so the optimization of the CNT composition for mechanical properties must be considered. A composite with a lower density and compatible mechanical properties was achieved via the simple melt blending of CF, CNT, PA6, and PP, and this material can be easily applied to lightweight automotive parts.

## Figures and Tables

**Table 1:** Summary of material mixing ratios.

Name of Composite	PA6/PP Blending (wt %)	CNT (wt %)	CF (wt %)
0% PP + 0.0% CNT	80.0 (100% PA6:0% PP:0% MaPP)	0.0	20
0% PP + 0.5% CNT	79.5 (100% PA6:0% PP:0% MaPP)	0.5	
0% PP + 1.0% CNT	79.0 (100% PA6:0% PP:0% MaPP)	1.0	
0% PP + 1.5% CNT	78.5 (100% PA6:0% PP:0% MaPP)	1.5	
10% PP + 0.0% CNT	80.0 (89.5% PA6:10% PP:0.5% MaPP)	0.0	
10% PP + 0.5% CNT	79.5 (89.5% PA6:10% PP:0.5% MaPP)	0.5	
10% PP + 1.0% CNT	79.0 (89.5% PA6:10% PP:0.5% MaPP)	1.0	
10% PP + 1.5% CNT	78.5 (89.5% PA6:10% PP:0.5% MaPP)	1.5	
20% PP + 0.0% CNT	80.0 (79.0% PA6:20% PP:1.0% MaPP)	0.0	
20% PP + 0.5% CNT	79.5 (79.0% PA6:20% PP:1.0% MaPP)	0.5	
20% PP + 1.0% CNT	79.0 (79.0% PA6:20% PP:1.0% MaPP)	1.0	
20% PP + 1.5% CNT	78.5 (79.0% PA6:20% PP:1.0% MaPP)	1.5	
30% PP + 0.0% CNT	80.0 (68.5% PA6:30% PP:1.5% MaPP)	0.0	
30% PP + 0.5% CNT	79.5 (68.5% PA6:30% PP:1.5% MaPP)	0.5	
30% PP + 1.0% CNT	79.0 (68.5% PA6:30% PP:1.5% MaPP)	1.0	
30% PP + 1.5% CNT	78.5 (68.5% PA6:30% PP:1.5% MaPP)	1.5	

**Table 2:** Process parameters for a single screw extruder and injection molding machine for specimen preparation.

Process	Parameter	Value
Single screw extruder	Temperature (°C)	180-250-250-230-230
	Rotation speed (RPM)	300
Injection molding machine	Temperature (°C)	260-275-285
	Injection pressure (kg/cm^2^)	100
	Clamping force (tons)	15
	Holding time (s)	20
	Volumetric rate (cm^3^/s)	5

**Table 3:** Mechanical properties and densities of CF/PA6 composites with different polypropylene (PP) ratios.

Name of Composite	Tensile Strength (MPa)	Elastic Modulus (GPa)	Flexural Strength (MPa)	Flexural Modulus (GPa)	Density (g/cm^3^)
0% PP + 0.0% CNT	200 ± 4	11.08 ± 0.44	275 ± 2	10.71 ± 0.33	1.214 ± 0.002
10% PP + 0.0% CNT	177 ± 4	10.22 ± 0.36	247 ± 3	9.40 ± 0.20	1.187 ± 0.003
20% PP + 0.0% CNT	171 ± 2	9.48 ± 0.08	236 ± 6	8.99 ± 0.21	1.160 ± 0.002
30% PP + 0.0% CNT	168 ± 2	9.46 ± 0.20	219 ± 2	8.57 ± 0.12	1.131 ± 0.002

**Table 4:** Comparison of mechanical properties and densities of CF-reinforced PA6/PP composites without CNTs and with CNTs.

Name of Composite	Tensile Strength (MPa)	Elastic Modulus (GPa)	Flexural Strength (MPa)	Flexural Modulus (GPa)	Density (g/cm^3^)
0% PP + 0% CNT	200 ± 4	11.08 ± 0.44	275 ± 2	10.71 ± 0.33	1.214 ± 0.002
10% PP + 1% CNT	196 ± 6	11.07 ± 0.29	273 ± 4	10.95 ± 0.29	1.199 ± 0.004
10% PP + 0% CNT	177 ± 4	10.22 ± 0.36	247 ± 3	9.40 ± 0.20	1.187 ± 0.003
20% PP + 0.5% CNT	179 ± 3	10.28 ± 0.21	270 ± 2	10.36 ± 0.27	1.163 ± 0.005
20% PP + 0% CNT	171 ± 2	9.48 ± 0.08	236 ± 6	8.99 ± 0.21	1.160 ± 0.002
30% PP + 0.5% CNT	173 ± 3	10.11 ± 0.19	239 ± 4	9.63 ± 0.26	1.135 ± 0.003
30% PP + 0.0% CNT	168 ± 2	9.46 ± 0.20	219 ± 2	8.57 ± 0.12	1.131 ± 0.002
